# Correlation between Cancer Stem Cells, Inflammation and Malignant Transformation in a DEN-Induced Model of Hepatic Carcinogenesis

**DOI:** 10.3390/cimb44070198

**Published:** 2022-06-29

**Authors:** Chun-Chieh Wu, Chien-Ju Lin, Kong-Kai Kuo, Wan-Tzu Chen, Chen-Guo Ker, Chee-Yin Chai, Hung-Pei Tsai, Sheau-Fang Yang

**Affiliations:** 1 Department of Pathology, Faculty of Medicine, Kaohsiung Medical University, Kaohsiung City 807, Taiwan; lazzz.wu@gmail.com (C.-C.W.); ccjtsai@yahoo.com (C.-Y.C.); 2 Department of Pathology, Kaohsiung Medical University Hospital, 100 Tzyou 1st Road, Kaohsiung City 807, Taiwan; wanzi@cc.kmu.edu.tw; 3 School of Pharmacy, College of Pharmacy, Kaohsiung Medical University, Kaohsiung City 807, Taiwan; mistylin@kmu.edu.tw; 4 Division of Hepatobiliary Surgery, Department of Surgery, Kaohsiung Medical University Hospital, Kaohsiung City 807, Taiwan; kuoksfo168@gmail.com; 5 Department of Surgery, Yuan’s General Hospital, Kaohsiung City 807, Taiwan; kercg@yuanhosp.com.tw; 6 Division of Neurosurgery, Department of Surgery, Kaohsiung Medical University Hospital, Kaohsiung City 807, Taiwan

**Keywords:** diethylnitrosamine, inflammation, hepatocellular carcinoma

## Abstract

Chronic inflammation and cancer stem cells are known risk factors for tumorigenesis. The aetiology of hepatocellular carcinoma (HCC) involves a multistep pathological process that is characterised by chronic inflammation and hepatocyte damage, but the correlation between HCC, inflammation and cancer stem cells remains unclear. In this study, we examined the role of hepatic progenitor cells in a mouse model of chemical-induced hepatocarcinogenesis to elucidate the relationship between inflammation, malignant transformation and cancer stem cells. We used diethylnitrosamine (DEN) to induce liver tumour and scored for H&E and reticulin staining. We also scored for immunohistochemistry staining for OV-6 expression and analysed the statistical correlation between them. DEN progressively induced inflammation at week 7 (40%, 2/5); week 27 (75%, 6/8); week 33 (62.5%, 5/8); and week 50 (100%, 12/12). DEN progressively induced malignant transformation at week 7 (0%, 0/5); week 27 (87.5%, 7/8); week 33 (100%, 8/8); and week 50 (100%, 12/12). The obtained data showed that DEN progressively induced high-levels of OV-6 expression at week 7 (20%, 1/5); week 27 (37.5%, 3/8); week 33 (50%, 4/8); and week 50 (100%, 12/12). DEN-induced inflammation, malignant transformation and high-level OV-6 expression in hamster liver, as shown above, as well as applying Spearman’s correlation to the data showed that the expression of OV-6 was significantly correlated to inflammation (*p* = 0.001) and malignant transformation (*p* < 0.001). There was a significant correlation between the number of cancer stem cells, inflammation and malignant transformation in a DEN-induced model of hepatic carcinogenesis in the hamster.

## 1. Introduction

Hepatocellular carcinoma (HCC) is a common malignancy that affects nearly one million people globally each year, but treatment options are limited [1]. The aetiology of HCC involves a multistep pathological process that is characterised by chronic inflammation and hepatocyte damage [2]. The dedifferentiation of mature liver cells has been implicated in HCC [3], based on which it was hypothesised that HCC arises from maturation arrest in liver stem cells and hepatic progenitor cells [4]. In light of this evidence, in this study, we examined the role of hepatic progenitor cells in a mouse model of chemical-induced hepatocarcinogenesis.

Cancer stem cells are tumour cells with the ability to self-replicate and differentiate into solid tumours, including HCC [3], and express known stem cell markers, such as CD133 in glioma, CD44 and CD24 in breast cancer and OV-6 in HCC [5,6,7].

Oval cells are associated with the intrahepatic biliary system and are derived from hepatoblasts located near hepatic portals. These cells and their progeny have the ability to proliferate and differentiate into either biliary cells or hepatocytes. In the mature liver, oval cells induce the replication of hepatocytes and cholangiocytes [8]. Oval cells have been demonstrated to arise in rat hepatic intraportal regions after treatment with hepatocarcinogens or hepatotoxins [8,9]. The cellular protein, OV-6, has been found to be a useful marker of rat oval cells, thought to be the progeny of hepatic stem cells [10,11]. The intraperitoneal injection of mice with diethylnitrosamine (DEN) was shown to induce tumours in the liver, gastrointestinal tract, skin and respiratory tract, as well as haematopoietic cancer [12,13,14]. In this study, DEN was used as a carcinogen in an experimental hamster model to induce liver tumour and determine the relationship between malignant transformation, inflammation and the role of cancer stem cells in the pathogenesis.

## 2. Materials and Methods

### 2.1. Animals

Male hamsters (*n* = 40) (LASCO; Taipei; Taiwan) were housed in plastic cages with soft bedding under a 12 h reversed light–dark cycle (light cycle from 6 a.m. to 6 p.m.; dark cycle from 6 p.m. to 6 a.m.) and access to food and water ad libitum. All experimental procedures were approved by the Kaohsiung Institutional Animal Care and Use Committee.

### 2.2. DEN-Induced Liver Cancer Model

The DEN-induced liver tumour model was established as described previously [12,13,14]. The hamsters were fed DEN (80 μg/g body weight/day) (Sigma-Aldrich; St. Louis; USA). The animals were sacrificed at various timepoints in the study (weeks 7, 27, 33 and 50), and the liver was removed and fixed in 4% paraformaldehyde and embedded in paraffin.

### 2.3. Histology Analysis

**Figure 1:**
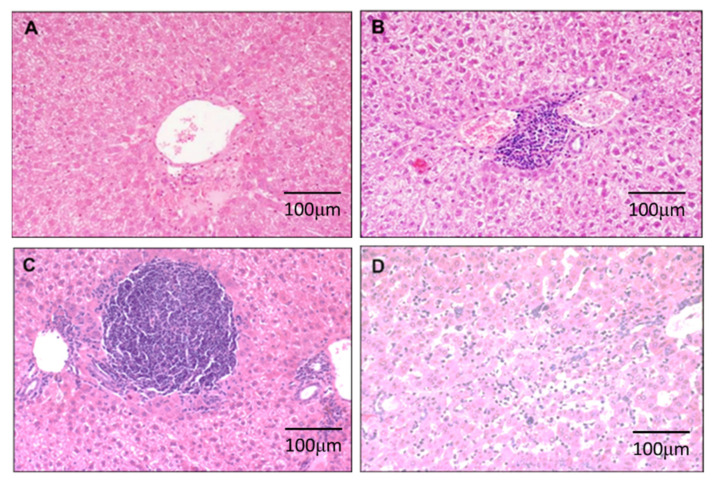
H&E staining of hamster liver: Portal inflammation was scored from 0 to 3 in week 7 (*n* = 5), 27 (*n* = 8), 33 (*n* = 8), 50 (*n* = 12) where a score of 0 indicated no portal inflammation (**A**); a score of 1 indicated ≤2 portal inflammation (**B**); a score of 2 indicated >2 portal inflammation (**C**); and a score of 3 indicated portal inflammation with interstitial sinusoidal inflammation (**D**).

Tissue sections (4 μm) were stained with haematoxylin and eosin (H&E). Each pathologist initially reviewed all the slides and reached an agreement on the scores. Portal inflammation was scored from 0 to 3 where a score of 0 indicated no portal inflammation (Figure 1A); a score of 1 indicated ≤2 portal inflammation (Figure 1B); a score of 2 indicated >2 portal inflammation (Figure 1C); and a score of 3 indicated portal inflammation with interstitial sinusoidal inflammation (Figure 1D).

### 2.4. Reticulin Staining

**Figure 2:**
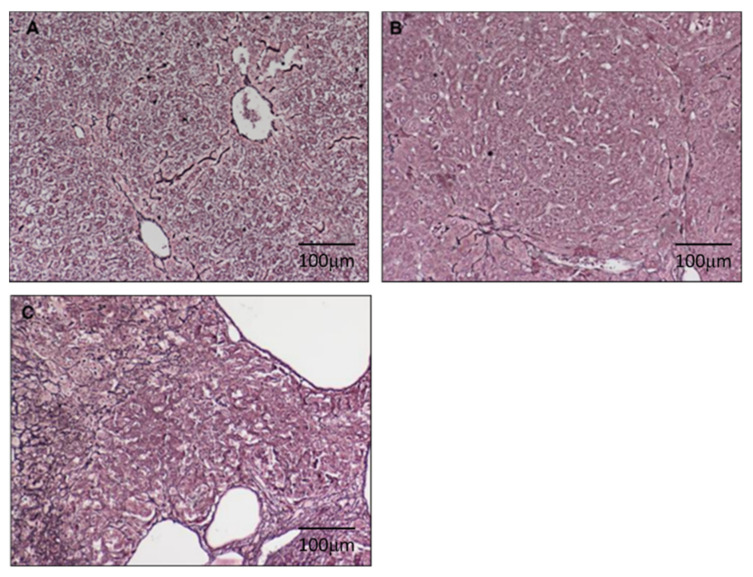
The reticulin staining in hamster liver: we applied a score ranging from 0 to 2 in week 7 (*n* = 5), 27 (*n* = 8), 33 (*n* = 8), 50 (*n* = 12) where a score of 0 indicated no regenerated/malignant cell (**A**); a score of 1 indicated the presence of liver regenerated nodules (**B**); and a score of 2 denoted definitive liver malignancy (**C**).

Tissue sections measuring 4 µm that were obtained after deparaffinisation and rehydration from paraffin-embedded samples were rinsed in distilled water and immersed in 1% potassium permanganate (Merck; Darmstadt; Germany) for 2 min, followed by treatment as follows: 2.5% oxalic acid (Merck; Darmstadt; Germany) for 1 min; 2% iron alum (Merck; Darmstadt; Germany) for 1 min; Gomori’s solution (Merck; Darmstadt; Germany) for 3 min; 10% formalin (Merck; Darmstadt; Germany) for 2 min; gold chloride (Merck; Darmstadt; Germany) (1:500) for 3 min; 3% potassium metabisulfite (Merck; Darmstadt; Germany) for 1 min; and 3% sodium thiosulfate (Merck; Darmstadt; Germany) for 1 min. Slide sections were rinsed with distilled water before immersion in each solution. Finally, the issue sections were examined using a light microscope (Nikon) and photographed; the images were saved as jpg files. For malignant transformation, we applied a score ranging from 0 to 2, where a score of 0 indicated no regenerated/malignant cell (Figure 2A); a score of 1 indicated the presence of liver regenerated nodules (Figure 2B); and a score of 2 denoted definitive liver malignancy (Figure 2C).

### 2.5. Immunohistochemistry

**Figure 3:**
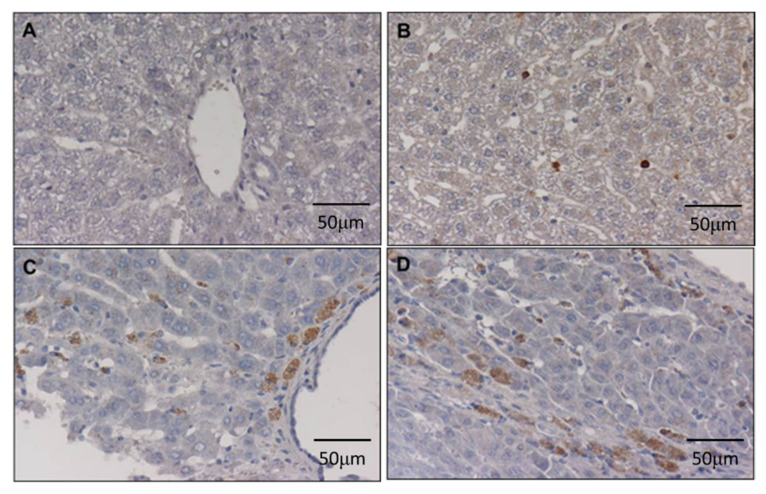
The immunohistochemical staining of OV-6 in hamster liver in week 7 (*n* = 5), 27 (*n* = 8), 33 (*n* = 8), 50 (*n* = 12): a score of 0 indicated no positive cells (**A**); a score of 1 indicated ≤5 stem cells in one high power field (**B**); a score of 2 indicated ≥5 and <10 stems cells in one power field (**C**); a score of 3 indicated >10 stem cells in one power field (**D**). Scores 0 and 1 were considered low-level expression, whereas scores 3 and 4 were considered high-level expression.

We obtained 3 μm tissue sections from paraffin-embedded samples that were subjected to deparaffinisation and rehydration. Following antigen retrieval by autoclaving for 5 min in DAKO antigen retrieval solution (DAKO, Carpinteria, CA, USA), the sections were washed twice in TBS buffer. Endogenous peroxidase was blocked by immersing the slides in 3% hydrogen peroxide solution for 5 min. After washing with TBS, the slides were incubated with primary antibody against OV-6 (mouse anti-OV-6; MAB2020; R&D; State of Minnesota; USA; 1:200) for 1 h at room temperature, subsequently washed two times with TBS and incubated with biotinylated secondary antibody for 30 min. After washing two times with TBS, the sections were treated with DAB for 5 min. Immediately after staining, the sections were counterstained with haematoxylin for 90 s, immersed in xylene and mounted using Permount (Fisher Scientific, Pittsburg, PA, USA). The sections were examined using a light microscope (Nikon) and then photographed; the images were saved as jpg files. The OV-6-immunostained tissue sections were scored as follows: a score of 0 indicated no positive cells (Figure 3A); a score of 1 indicated ‘≤5 stem cells in one high power field’ (Figure 3B); a score of 2 indicated ‘≥5 and <10 stems cells in one power field’ (Figure 3C); and a score of 3 indicated ‘>10 stem cells in one power field’ (Figure 3D). Scores 0 and 1 were considered low-level expression, whereas scores 3 and 4 were considered high-level expression.

### 2.6. Data Analyses

SPSS 24.0 (IBM, Armonk, NY, USA) software was used for the statistical analysis. The relationships between the score of malignant transformation, inflammation and OV-6 were determined using Spearman’s correlation test. Statistical significance was considered when *p* < 0.05.

## 3. Results

### 3.1. DEN-Induced Inflammation in Liver

Inflammation was determined by examining the scores that were obtained in the H&E-stained sections (Figure 1). We found that DEN progressively induced inflammation at week 7 (40%, 2/5); week 27 (75%, 6/8); week 33 (62.5%, 5/8); and week 50 (100%, 12/12).

### 3.2. DEN Induces Malignant Transformation in the Liver

Malignant transformation was determined by examining the scores that were obtained from the reticulin-stained sections (Figure 2). We found that DEN progressively induced malignant transformation at week 7 (0%, 0/5); week 27 (87.5%, 7/8); week 33 (100%, 8/8); and week 50 (100%, 12/12).

### 3.3. DEN Enhances Cancer Stem Cell Marker in the Liver

The cancer stem cells were identified based on the immunohistochemical expression of the biomarker, OV-6 (Figure 3). The obtained data showed that DEN progressively induced high-levels of OV-6 expression at week 7 (20%, 1/5); week 27 (37.5%, 3/8); week 33 (50%, 4/8); and week 50 (100%, 12/12).

### 3.4. Relationship between Cancer Stem Cell, Inflammation and Malignant Transformation

**Figure 4:**
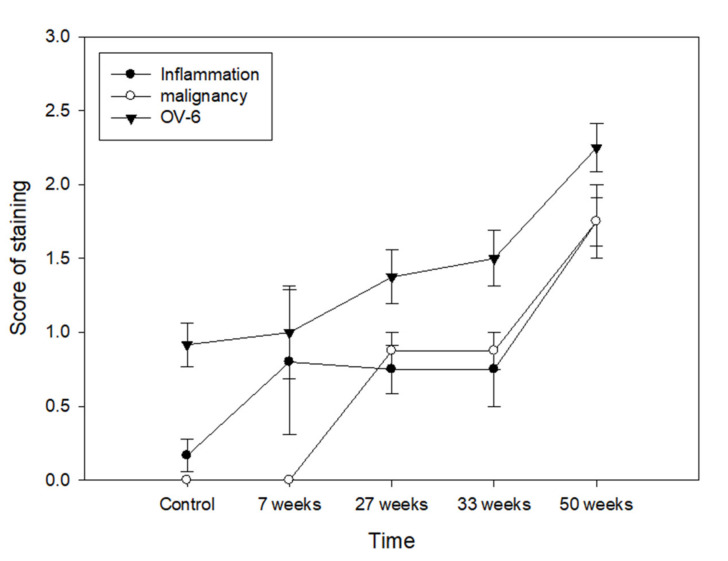
Correlation between malignant transformation, cancer stem cells and inflammation. *p* value was calculated by Spearman’s correlation test.

DEN-induced inflammation, malignant transformation and high-level OV-6 expression in hamster liver, as shown above, and applying Spearman’s correlation to the data showed that the expression of OV-6 was significantly correlated to inflammation (*p* = 0.001) and malignant transformation (*p* < 0.001) (Figure 4).

## 4. Discussion

The goal of this study was to determine the relationship between malignant transformation, inflammation and the role of cancer stem cells in the pathogenesis of HCC. In order to do so, we used DEN as a carcinogen in an experimental hamster model to induce liver tumour, and in this study, we showed that the expression of OV-6 was significantly correlated to inflammation and malignant transformation in the DEN-induced hamster model of carcinogenesis.

Liver cancer is the third leading cause of cancer death and the fifth most common cancer worldwide [15]. Because the mechanisms of pathogenesis are not clearly known, the therapeutic approaches for hepatic malignancies are limited. To elucidate this further, the possible mechanisms inducing human HCC are classified into four groups: growth factors and their receptors (TGF-α) [16,17]; the reactivation of developmental pathways (Wnt) [18]; oncogenes (K-ras) [19]; and tumour suppressor gene (p53) [20,21].

Cancer stem cells can self-renew, proliferate, metastasise, cause relapse and induce resistance to chemotherapy and radiation therapy [22]. Cancer stem cells have been identified in various human cancers, including that of the breast [23], colon [24], prostate [25], pancreas [26], as well as in head and neck squamous cell carcinoma [27]. We showed in this study that DNE-induced HCC expressed more OV-6 positive cells than in control animals, indicating the increased presence of cancer stem cells in a DEN-induced model of HCC.

Inflammation is associated with an increased risk for various malignant neoplasms, carcinogenesis, metastasis, angiogenesis, tumour invasion, anti-apoptosis, epigenetic modifications, genomic instability, enhanced cell proliferation and aggressive tumour neovascularization [28,29,30]. Immune cells including lymphocytes and macrophages, platelets, fibroblasts and tumour cells are a major source of angiogenic factors [31,32,33], which play an essential role in leukocyte infiltration into the tumour microenvironment, thereby regulating the tumour size, distribution and composition. Indeed, tumour-associated macrophages are key regulators of the link between inflammation and carcinogenesis [34,35].

The activation of hepatic progenitor cells in chronic hepatitis C infection is a common occurrence that depends on the hepatitis stage [36]. Stem cell numbers increase with sinusoidal or interstitial inflammation, and particularly in HCC, they are located within liver tumours and scattered as single cells, not in the portal tracts, bile ducts or canals of Hering. These stem cells assume the morphology of their neighbouring hepatocytes in both cirrhosis and HCCs [37] and proliferate in fibrous areas and liver tumour parenchyma as the tumours develop. In the current study, reactive ductules and intermediate hepatocyte-like cells originated partly from the activation and differentiation of ‘progenitor cells’ in the hamster liver, whose proliferation is associated with an increase in inflammation or the damage of hepatocytes and the tumour status. Previous studies showed that the progression of HCC was found through the Wnt pathway, the loss function of p53, Ras signalling, and the ROS pathway [38]. Chronic inflammation induced the proliferation of hepatocytes, the shortening of telomeres and malignant transformation [39]. Moreover, the occurrence of repeated and continuous liver injury is significantly increased in advanced cirrhosis, leading to a propensity towards cancer and fibrosis [40]. Therefore, inflammation leads to fibrosis and malignant transformation.

Oval cells played an important role in the development of HCC [41]. OV-6 is a biomarker of oval cells and derives from hepatic stem cells [7,8]. In addition, OV-6 has a well-known association with prognosis in various tumours including HCC [42]. However, no reports showed that OV-6 was associated with inflammation or malignant transformation. In our result, DEN-induced inflammation, malignant transformation and high-level OV-6 expression in hamster liver was significantly correlated to inflammation and malignant transformation (Figure 4).

## 5. Conclusions

There was a significant correlation between the number of cancer stem cells, inflammation and malignant transformation in a DEN-induced model of hepatic carcinogenesis in the hamster. It is postulated that the cancer developed through an inflammatory process, which increased the cancer stem cells within the liver tumours. This study describes that repeatedly inflammatory responses induce malignant transformation and the development of cancer stem cells that are resistant to chemotherapy and radiotherapy.

## Data Availability

Not applicable.
